# Navigating Outdoor Activities During Heatwaves: Heat Resilience Insights from a Focus Group Study with Urban-Dwelling Older Adults in Sweden

**DOI:** 10.1007/s44474-026-00009-z

**Published:** 2026-11-05

**Authors:** Sofia Kvarnström Börsbo, Johanna Johansson, Lena Rosenberg, Susanne Gustafsson

**Affiliations:** 1 Department of Rehabilitation, Jönköping University, Jönköping, Sweden; 2 Swedish National Graduate School on Ageing and Health (SWEAH), Lund, Sweden

**Keywords:** Age-Friendly Cities and Communities, Climate change, Occupational therapy, Sustainable urbanization, Urban environments

## Abstract

**Purpose:**

Climate change is increasing the frequency and intensity of heatwaves, posing significant health risks for older adults. In Sweden, older adults have historically not needed to consider heat during outdoor activities, but rising temperatures make such considerations increasingly important. Urban environments with limited vegetation and high heat exposure exacerbate these risks and are restricting their participation in society. To promote Age-friendly Cities and Communities, more knowledge is needed about how heatwaves influence urban-dwelling older adults’ outdoor activities. Therefore, this study aimed to provide insight into how older adults reason about activities in urban outdoor environments during heatwaves.

**Methods:**

Data were generated from three focus groups involving a total of 19 older adults and analyzed using reflexive thematic analysis.

**Results:**

Four themes were created: individual adaptations to outdoor activities; the significance of social networks; society’s responsibility to act on the challenges posed by heatwaves; and opportunities and barriers in the urban outdoor environment. Outdoor activities were influenced by factors at individual, group, and societal levels. These included personal habits, social networks, and environmental opportunities and barriers.

**Conclusions:**

Heat resilience requires age-friendly urban planning and tailored preparedness strategies, grounded in a holistic understanding of older adults’ everyday lives. Supporting habit adaptation, identifying persons at risk of heat-related health impacts, and addressing high-risk environments are essential. Occupational therapists could assume an important preventive role that strengthens participation, autonomy, and resilience among older adults in a changing climate.

## Introduction

Climate change is expected to become one of humanity’s greatest threats, with negative consequences for public health [1]. As the climate changes, heatwaves will increase in frequency, intensity, and duration—both globally and in Sweden [2]. Historically, the population in Sweden has rarely needed to consider heat, as heatwaves have been uncommon so far in Nordic countries [3], and urban planning has traditionally been adapted to cold climates rather than for heat resilience [4]. There is no international definition of a heatwave, as it varies between geographical regions and local conditions. In a Nordic context, a heatwave is defined as five consecutive days where the highest daily temperature reaches at least 25 °C [5].

The Public Health Agency of Sweden [6] has identified heatwaves as the most significant climate-related health risk in Sweden, based on severity and likelihood. Vulnerable groups are at the greatest risk of adverse health effects, with older adults particularly vulnerable to heat stress [2]. Heat stress occurs when the body can no longer regulate temperature through sweating and increased blood flow, causing body temperature to rise and affecting respiration and circulation [7]. Older adults are considered a risk group due to impaired temperature regulation, a higher prevalence of chronic illnesses, and frequent use of medications that may have serious side effects in high temperatures [8]. According to a report from Statistics Sweden [9], one quarter of Sweden’s population is aged 60 years or older. The report emphasizes the importance of not viewing older adults as a homogeneous group, as there can be significant differences in age, background, health, housing, employment, income, activity capacity, and lifestyle. These factors influence not only participation in everyday activities but also how older adults experience and manage heatwaves.

The conditions for participation in society, wellbeing, and health are shaped by personal factors as well as by the environment. One of the UN’s global goals for 2030 is to create sustainable cities and communities (#11), which includes promoting inclusive and sustainable urbanization [10]. Age-Friendly Cities and Communities play a crucial role in promoting participation, wellbeing, and health among older adults, especially during climate-related challenges such as extreme heat. By designing environments that consider the needs of older adults, better conditions are created for everyday activities, benefiting both the individual and society as a whole. To become members of the WHO Global Network for Age-friendly Cities and Communities, cities and communities are expected to demonstrate political commitment, involve older adults, conduct local assessments, develop action plans, and engage in ongoing evaluation [11].

Today, 88% of Sweden’s population lives in urban areas [12]. Urban outdoor environments consist mainly of paved surfaces, which implies a risk for higher temperatures. Temperatures in urban areas are already generally higher compared to surrounding rural areas, particularly at night due to the urban heat island effect [13]. This makes people living in cities particularly vulnerable during heatwaves. Certain building materials act like radiators, causing cities to cool slowly and making it especially harder for vulnerable groups to recover at night. The denser the buildings, the greater the proportion of paved surfaces and the less vegetation, the stronger the heat island effect [7]. Characteristics of the built environment and other factors associated with population growth increase exposure to extreme heat and pose significant health risks [14].

Studies have shown that heatwaves affect older adults’ participation in society [4, 15], including hindering their continuation of outdoor activities. Lönn et al. [4] report that 95% of older adults avoided outdoor activities during urban heatwaves, with 39% always or almost always avoiding such activities. These findings indicate the importance of creating urban environments that support participation in society even under extreme weather conditions, particularly as outdoor activities are essential for older adults’ health and preventing social isolation [16].

How heatwaves affect an older adult is personal and, as previously mentioned, depends on various interacting elements. The Transactional Model of Occupation [17] describes how activities and contextual elements continuously shape one another, offering a holistic understanding of these dynamics. The model can support an understanding of how outdoor activities are performed, experienced, and foster participation for individuals, groups, or communities during heatwaves. It considers how current activities are influenced by client, temporal, sociocultural, geopolitical, physical environmental, social environmental, and task components. These components affect not only the activities themselves but also each other [17], highlighting the complexity of older adults’ activities in different contexts.

Building on the Transactional Model of Occupation [17], occupational therapy emphasizes the importance of understanding how interactions between individuals, occupations, and environments impact everyday life. This understanding is particularly important in the context of climate-related challenges such as heatwaves, which may alter opportunities for engagement in meaningful outdoor activities. To meet future challenges posed by heatwaves and create communities that support participation in society, wellbeing, and health, it is essential to understand the impact of heatwaves at a local contextual level [18]. There is limited knowledge of how older adults in a Nordic context describe their own experiences and thoughts regarding outdoor activities during heatwaves [15], both how they are affected today and their expectations for the future. This knowledge is required to understand the specific needs that emerge during heatwaves, the strategies to address these challenges effectively, and how the occupational therapy profession can support older adults in maintaining their engagement in meaningful activities, while contributing to the development of inclusive and sustainable urban environments for an aging population. Insights into urban-dwelling older adults’ own perspectives regarding outdoor activities during heatwaves can contribute to knowledge that is essential for health-promoting measures at the individual, group, and societal levels. Therefore, the aim of this study was to provide insight into how older adults reason about activities in urban outdoor environments during heatwaves.

## Methods

A focus group methodology was chosen to highlight the collective perspective of older adults’ reasoning about activities in urban outdoor environments during heatwaves. Focus groups were considered particularly appropriate for capturing breadth and variation in the reasoning, as well as the ways in which participants compare, negotiate, and build on each other’s perspectives in interaction [19, 20]. In line with a social constructivist perspective, the method recognizes knowledge as co-created through social interaction. This was relevant to the present study, as activities in urban outdoor environments during heatwaves are shaped not only by individual capacities and preferences, but also by shared norms, social networks, and characteristics of the urban environment. The group format therefore enabled participants to exchange experiences, reflect on similarities and differences, and generate nuanced reasoning about both opportunities and barriers in activities in urban outdoor environments during heatwaves. This method also provided an opportunity to explore their reasoning in depth, including the nuances in participants’ perspectives that emerged from their group interactions [19, 20].

### Researcher Positioning and Reflexivity

The authors shared background as occupational therapists and a shared occupational science perspective shaped the focus on activities and environmental conditions. All authors have experience of qualitative research, and the third and fourth authors have in-depth methodological knowledge in conducting focus group studies. Acknowledging that qualitative findings are shaped by researchers’ interpretations, the authors addressed potential bias through reflexive discussions, examination of alternative interpretations, and collaborative revisiting of analytical decisions to strengthen transparency and trustworthiness.

### Participants

Inclusion criteria for participants were persons aged 65 or older, residing in and moving about in urban outdoor environments, and able to speak and understand Swedish. No exclusion criteria were applied. A balance between homogeneity and heterogeneity in the focus groups was sought to create a permissive conversational climate and achieve broad representation of the target group [19]. Homogeneity referred to older adults sharing similar experiences of activities in urban outdoor environments during heatwaves, providing a common foundation for discussion and interaction within the focus group. Heterogeneity included variation in gender, age, type of housing, and municipality. These characteristics were used as guiding criteria during recruitment and group composition to support variation within the overall sample while maintaining a shared experiential basis within each focus group.

Participants were recruited through strategic and snowball sampling [21] in Jönköping and Gothenburg, Sweden. Recruitment was conducted through several channels in both municipalities to reach participants with shared relevance to the study aim while enabling variation in sociodemographic and contextual characteristics. Recruitment began via gatekeepers informing individuals about the study within their organizations, obtaining verbal consent for participation, and passing on contact information to the authors. Gatekeepers were municipal senior consultants, development leaders for Age-friendly Gothenburg, and the chair of the Senior University in Jönköping. Information about the study was also disseminated during the first author’s visits to meeting places for seniors in Jönköping, and through posters placed at meeting hubs in both Jönköping and Gothenburg.

Recruitment resulted in three focus groups—two in Jönköping and one in Gothenburg (Table 1). To allow for potential dropouts, groups of up to eight participants were aimed for, ultimately ranging from five to eight participants. A total of 22 individuals were recruited, of whom three withdrew due to illness, resulting in 19 participants.

**Table 1:** Sociodemographic data of the focus group participants

Focus group (number = no) (participants = p)	Gender (female/male)	Age (distribution, year (average, year)	Type of housing (apartment/townhouse/villa)	Employment (retired/part-time/full-time)	Municipality
no 1: *p* = 8	7/1	72–82; 79	7/0/1	7/1/0	Jönköping
no 2: *p* = 6	5/1	67–81; 74	5/1/0	3/2/1	Gothenburg
no 3: *p* = 5	5/0	71–93; 83	5/0/0	5/0/0	Jönköping
**Total (no 1–3): *p* = 19**	**17/2**	**67–93; 78**	**17/1/1**	**15/3/1**	

### Study Setting

The study was conducted in two Swedish municipalities, Gothenburg and Jönköping, which represent different urban and geographical contexts. Gothenburg is a larger coastal municipality on Sweden’s west coast, with approximately 613,000 inhabitants [22]. Its proximity to the sea contributes to a maritime climate [23]. In contrast, Jönköping is a medium inland municipality with approximately 148,000 inhabitants [22]. Located by Lake Vättern, Sweden’s second-largest lake, Jönköping has a more continental climate with lakeside characteristics [23]. Together, these municipalities provided contrasting settings that offered insight into older adults’ reasoning about activities in urban outdoor environments during heatwaves.

### Data Collection

All focus groups were conducted in person at local meeting hubs during February to March 2025, lasted approximately 2 h, and were led by the first and second authors. To create favorable conditions, each session began with a presentation about the study, clarifying the aim and the participants’ expected role [19]. The presentation also included images, newspaper headlines, and a short video to prompt memories and associations related to heatwaves in urban environments. During the introduction, written consent was obtained, along with sociodemographic background information. The focus groups were audio-recorded and transcribed verbatim by the first author, both manually and with the help of the transcription tool aTrain [24].

Discussion topics were based on a guide with open-ended questions, used as structural support for the moderator and to capture key aspects related to the study’s aim. The guide focused on three overarching domains: experiences of heatwaves, adaptations to outdoor activities, and conditions influencing activities in outdoor environments during heatwaves. The focus groups were led by a moderator whose task was to foster a permissive conversational climate. However, it was emphasized to participants that they themselves were the experts on the topic, not the moderator [20]. The domain experiences of heatwaves were introduced through the key question, “What are your experiences of performing outdoor activities during extreme heat and heatwaves?”, which served as the opening question in each focus group. Thereafter, the moderator adopted a flexible approach, allowing the issues raised in the participants’ discussions to inform the sequence and use of follow-up questions from the guide. For example, the question “Are there any strategies that have worked for you and that you could share with others?” addressed the domain adaptations to outdoor activities, whereas “If you had the opportunity to change something in your outdoor environment to improve your experience of activities, what would it be?” addressed the domain conditions influencing activities in outdoor environments during heatwaves. Although the questions varied according to the issues raised by participants, the moderator ensured that all three domains were addressed in each focus group. Each focus group also included an assistant moderator responsible for recording and taking field notes [19]. At the end of each session, the assistant moderator asked follow-up or clarifying questions and concluded by summarizing the discussion based on the field notes, giving participants the opportunity to respond.

Immediately after each focus group, the moderator and assistant moderator reflected on the outcome and key insights. These reflections included how participants responded to the key questions, the moderator’s role, and the perceived interaction and atmosphere within the group [19]. These debriefings were recorded and used as part of the reflexive approach in the analysis.

### Data Analysis

Reflexive thematic analysis according to Braun and Clarke [25] was used as the analytical method. It was considered suitable given the richness of the material generated in the focus groups and allowed interpretation of the open discussions. The analytical process was iterative and characterized by a reflexive approach.

An initial understanding of the content began to form already during the focus groups, and analysis continued with repeated listening to the recordings and multiple readings of the transcripts, both between and after the focus groups, to develop a deeper understanding of the content. The first author conducted the initial manual coding to identify patterns and potential differences between the focus groups during the early stages of thematization. Initially, the material was reviewed in relation to the three overarching areas from the question guide (experiences, adaptations, and conditions for activities in urban outdoor environments during heatwaves) to support familiarization with the data and to ensure that the discussion addressed the study aim and covered a broad range of perspectives. The thematization was developed gradually, from a general structure to more specific and in-depth themes, as the understanding of the material deepened. Throughout this process, the emerging codes and themes were continuously discussed with the co-authors, who reviewed and compared them with the data to ensure that they reflected the overall content and aligned with the study’s aim. As part of the quality review, recordings were re-listened to and transcripts re-read with a focus on the identified themes. In the final stage, the themes were refined and clarified further, and representative excerpts from the discussions were selected to illustrate and support the thematization. The results were written as part of the reflexive and iterative analysis, during which the themes were further refined through the writing process.

## Results

**Fig. 1:**
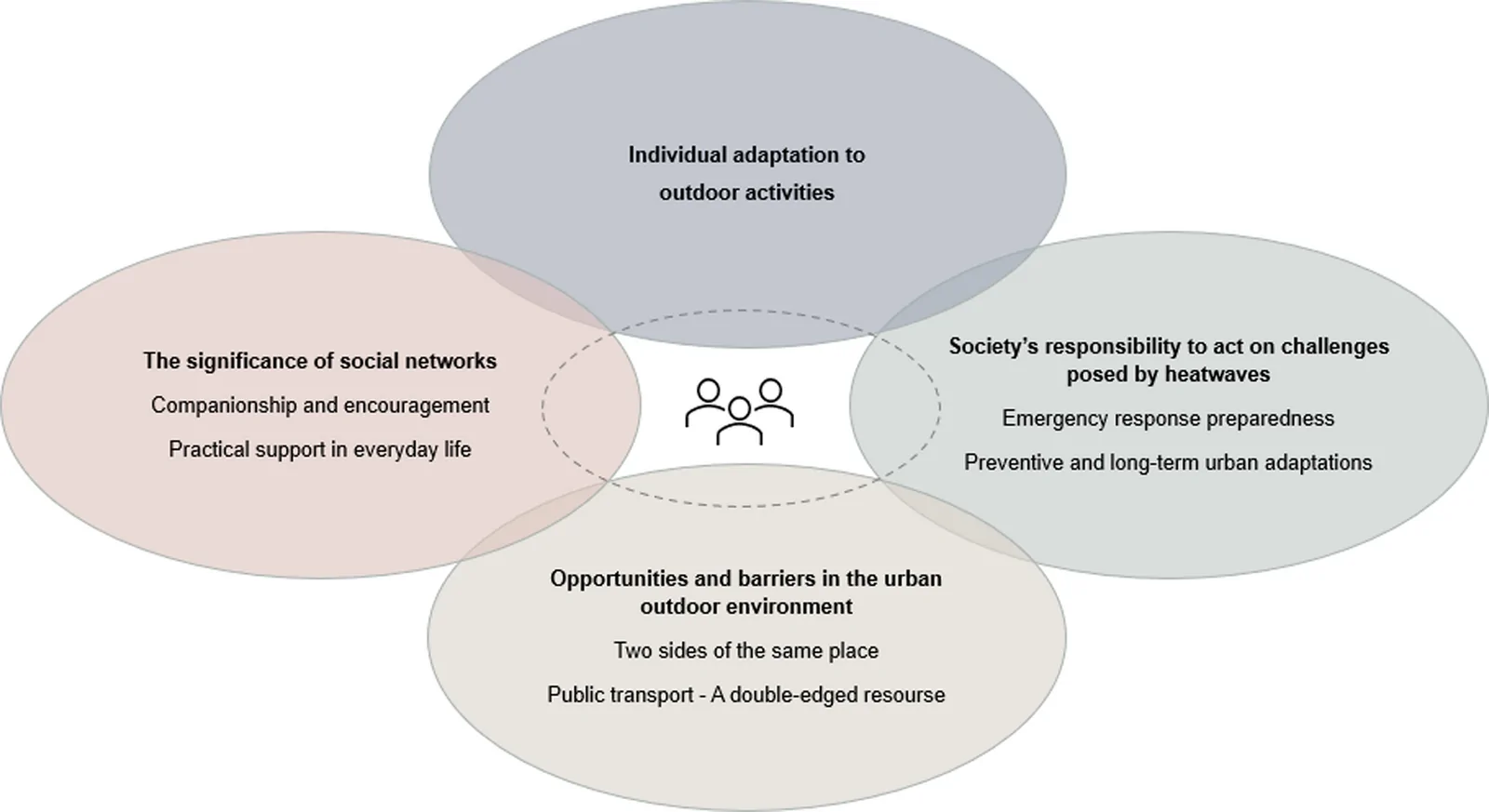
Illustration of urban-dwelling older adults’ reasoning about outdoor activities during heatwaves. The themes should not be viewed in isolation but understood as dynamically interacting, where variations in personal and environmental conditions constitute central aspects

The analysis resulted in four themes with corresponding subthemes that collectively reflect how older adults reason about activities in urban outdoor environments during heatwaves (Fig. 1). The four themes were as follows: *Individual adaptations to outdoor activities*, *The significance of social networks*, *Society’s responsibility to act on challenges posed by heatwaves*, and *Opportunities and barriers in the urban outdoor environment*. These themes should not be viewed as independent of one another but rather understood as dynamically interacting.

### Individual Adaptations to Outdoor Activities

Reasoning about outdoor activities during heatwaves highlighted the importance of individual adaptations. These centered particularly around personal routines: the ability to maintain them and the need to temporarily adjust them. The need for and strategies of adaptation varied depending on physical and cognitive capacity, access to resources, housing conditions, and personal preferences. For instance, having access to a summer cottage enabled some to temporarily leave the city, though this was only feasible as long as the energy required to manage the property could be sustained. At the same time, this option also reflected inequalities, since not everyone had the resources or opportunities to escape the urban heat. Housing design also shaped how heat was managed, including indoor temperature and access to a balcony or outdoor space. When adaptation was not feasible, refraining from outdoor activities appeared to be a strategy, although often accompanied by concerns about passivity and low mood. Routines emerged as important for promoting health, such as daily walks and maintaining social engagement. At the same time, heatwaves often required changes in routines, which were neither obvious nor easy for everyone. Adaptations could involve going out in the early morning or late evening, which were discussed as challenging what they perceived as the prevailing ‘Swedish norm’. This reasoning was elaborated in one discussion:

I don’t think one should be outdoors when it’s that hot. I believe that kind of change is necessary - that we become limited, that we have to accept that it’s like that on certain days, and try to adjust a bit. We could learn from other cultures that don’t engage in much activity between noon and 4 p.m. (group agrees)

I agree. If you look at how older and more senior people move about during the winter months, it’s during daylight hours. People don’t usually go out when it’s cold or dark. So you could just as well say that in summer, you avoid going out when it’s hot and instead go out in the late afternoon or evening. That’s an adaptation, and if we’re talking about values, I don’t see that as a disadvantage.

That you adapt?

If you look at the population, sure, there are people our age who go out in the evening. But most tend to avoid going out when it’s dark, especially at night. So we’re constantly adapting to our environment. And why would that be seen as a disadvantage? Staying in the shade, staying indoors during the hottest part of the day, and using the evenings instead - I don’t see the problem.

It’s a bit like… we should embrace Mediterranean culture more, be out in the evenings.

(Focus group 2)

These reflections illustrated how adaptations were not only framed as a practical necessity but also as a cultural shift, where participants questioned prevailing norms and drew inspiration from southern European practices. Strategies identified included seeking shaded walking paths or using public transport instead of walking. Indoor walking in places such as shopping malls appeared to be a clever alternative when outdoor temperatures were too high. Heat was also associated with increased fatigue, which created concerns about reduced energy and made it more difficult to initiate social activities during summer months when many services are closed.

Personal sun protection was discussed, although many found it difficult to get used to wearing hats, sometimes even with a touch of humor about how older adults struggle with them. In this context, strategies like using umbrellas or cooling garments appeared to be alternatives that enabled protection against the heat. These were not common practices today and could be seen as rather innovative suggestions, at least in the Swedish context. In some cases, the intensity of the heat made it impossible to be outdoors, prompting everyday adaptations such as shopping online or relying on home care services. These adaptations, however, raised concerns about digital competence and feelings of reduced control over product choices.

Overall, a tension emerged between flexibility and security, where the desire to maintain familiar routines was weighed against the need to adapt to feel well. Establishing healthy habits early in life was emphasized as important, since changing routines becomes more challenging with age. Such habits were considered essential for managing heatwaves in the long term.

### The Significance of Social Networks

Older adults’ vulnerability to social isolation and heightened dependence on their surroundings during heatwaves was a recurring theme in the discussions. Within this theme, their social network emerged as invaluable. Their social networks were described both as a source of *Companionship and encouragement* for engaging in outdoor activities and as a means of receiving *Practical support in everyday life*.

#### Companionship and Encouragement

Heatwaves were perceived to affect older adults both physically and psychologically, increasing the risk of isolation and inactivity. Encouragement within social networks was emphasized as a central way of sustaining interaction and occupations during periods of extreme heat. Encounters with others functioned as a strong motivator for going outside despite the challenging conditions. The notion of not “losing the social aspect of life” was emphasized as essential to wellbeing, with conversation and mutual motivation identified as key components. Several participants drew comparisons between heatwaves and their experiences during the COVID-19 pandemic, noting similar difficulties in initiating activities and an increased reliance on social support. Summers, particularly July, emerged as a period marked by disappointment and frustration, as organized activities paused and many people were away on vacation, leaving those living alone at greater risk of involuntary loneliness.

#### Practical Support in Everyday Life

The importance of mutual assistance was highlighted as central during heatwaves. A sense of responsibility emerged among those who considered themselves more resilient, often accompanied by pride in being able to support peers who were perceived as more vulnerable. Social networks were portrayed as essential, providing older adults with the confidence and reassurance to ask for practical support, such as assistance with shopping or reminders to stay hydrated.

Neighborhood cooperation emerged as a concrete and accessible form of support. Suggestions included posting notes in stairwells with offers of help or invitations to shared meals. Engagement in community organizations was also emphasized as a way of providing practical support to other older adults, particularly during periods of extreme heat. This reasoning was elaborated in one discussion:

Perhaps a companion, maybe. I’m not sure, but - like during the pandemic - the Tenants’ Association in Gothenburg stepped in right away, posting notices in all public housing buildings asking if people could help their neighbors with shopping and things like that. It was really great. Maybe we should have something similar during heatwaves.

Great idea! (agreement from several group members)

The Red Cross or the Tenants’ Association - those of us involved are very proactive and happy to help. Maybe someone could accompany those who want to go out for a short walk, so they don’t have to go alone.

Yes, great idea! (agreement from several group members)

A companion service.

Yes, a companion service. Beautiful term.

I think it already exists, actually. A companion service.

Then we’ll take that idea to the Red Cross headquarters and tell them to start a heat… (laughter)

Heat service. (laughter)

(Focus group 2)

These reflections illustrate how participants envisioned practical support as both neighborly and organized, drawing inspiration from pandemic responses while also introducing creative ideas such as “companion service” or even a light-hearted suggestion of a “heat service.” The exchange highlights how solidarity was intertwined with humor and pride, reinforcing the notion that everyday cooperation could be mobilized to protect older adults during heatwaves.

### Society’S Responsibility to Act on Challenges Posed by Heatwaves

Participants reflected on society’s substantial responsibility to act on challenges associated with heatwaves. Their reasoning emphasized both the need for *Emergency response preparedness*, requiring timely and coordinated action when a heatwave occurs, and the importance of *Preventive and long-term urban adaptations* that safeguard the health and wellbeing of older adults.

#### Emergency Response Preparedness

Participants highlighted the urgent need for societal preparedness to care for older adults and enable outdoor activities during heatwaves. Such reflections conveyed a strong sense of urgency, as participants framed preparedness not as a distant policy issue but as an immediate necessity, comparable to preparedness for war. It should be included in a comprehensive plan, and that timely implementation was crucial.

Yes, heat planning. I mean, they’re good at planning for war and such, but this is part of the climate war against us.

Absolutely, societal preparedness.

Definitely, definitely.

We’ve said several times that there should be some kind of preparedness plan where all [emphasis on all] the things we’ve discussed here are included - and perhaps it’s the responsibility of the Swedish Civil Defence and Resilience Agency to develop it.

But it’s also important to do it in time, because if you’re going to set up these portable setups, there are people who need to do it - and then you need to act two or three days in advance.

(Focus group 2)

Access to cool environments was emphasized as essential, with open public indoor spaces such as shelters or underground facilities being mentioned, sometimes with a mix of seriousness and humor, as places where individuals might seek relief. Participants also envisioned measures such as increased boat tours in waterfront cities and temporary shading structures, such as sun sails or parasols, at seating areas and pedestrian zones. Cars were perceived as heat-generating, and traffic bans in certain urban areas during heatwaves were framed as a strategy to improve conditions for outdoor activity.

Water resources emerged as a central aspect for older adults to manage heat and sustain outdoor activities, both to cool the body and to prevent heat-related symptoms such as swollen legs or cardiac discomfort. Access to outdoor showers, water hoses, ponds, and refill stations for water bottles was described as important, while also evoking memories of childhood urban environments. This connection situated adaptations within a cultural narrative of loss of communal resources, particularly in cities lacking access to lakes or the sea.

Proposals for adapting public services during heatwaves reflected participants’ awareness of how everyday activities become more challenging. Physician home visits and expanded access to home care services were highlighted as key measures. Support from volunteer youth was also discussed, with participants sharing mixed experiences of positive encounters and frustration over past situations where interactions felt dismissive or lacking in respect. This variation underscored the perceived importance of training to ensure that youth involvement genuinely supports older adults in ways that feel dignified and empowering. Parallels were drawn to the COVID-19 pandemic, where designated shopping hours for older adults served as a reference point for imagining future preparedness.

A recurring pattern concerned the need for clear and targeted information from public authorities ahead of heatwaves. Participants requested tailored warnings, recommendations, and practical advice regarding clothing, hydration, cooling locations, and risk areas. To ensure broad outreach, they emphasized dissemination through multiple channels, including television, radio, text messages, brochures, and digital displays in public transport and urban spaces.

#### Preventive and Long-Term Urban Adaptations

Participants reflected on the need for cities to be planned with heat in mind, not solely oriented toward a cold climate, and suggested that society should draw inspiration from southern European countries, which have greater experience in managing heatwaves. A desire emerged to establish a dedicated public actor responsible for strategic and long-term urban planning for a warmer climate, envisioned as a collaborative arena where older adults could contribute alongside policymakers. This reflected participants’ frustration that political influence today is often dominated by younger voices, leaving older adults feeling sidelined in decisions that directly affect their everyday lives. Despite the availability of knowledge on age-friendly urban development, participants also expressed frustration regarding the lack of concrete action. Participants’ ideas included heat-insulated housing with green rooftops and walls to provide both cooling and water retention, as well as an increase in shaded green areas. Current urban planting was seen as disappointing, as it tends to favor shrubs and small trees, while large trees—capable of providing shade and increasing humidity—were notably absent. Participants argued that material choices in urban environments should be reconsidered, noting that asphalt and concrete retain heat and should therefore be avoided. Some also envisioned a risk classification system for housing, based on its adaptability to heat. This long-term urban planning was vividly expressed and framed as a political issue:

But I think we really need to consider how we plan the city.

Exactly.

They say it’s extremely expensive to plant trees in the city as it is today. It’s not really affordable, so it only happens in new developments. When planning a new district, it’s there [emphasis on there] that we need to be careful - both with how buildings are positioned to allow for good airflow between them, and with ensuring there’s plenty of greenery. (group agrees)

Green rooftops, like growing things on rooftops, for example.

It’s been trendy among architects and urban developers to densify the city, but now there’s a counter-movement, as I understand it - people are trying to create more green spaces, realizing the impact of climate emissions and also heatwaves. So, a halt on densification, if we look at the politicians up there.

What you’re saying is that we also need to think long-term. And with new developments, there’s now the opportunity and the budget to plant a lot of greenery, a lot of trees when building new areas. So do it. Plant as much as possible in all new neighborhoods. That’s at least a long-term plan. (group agrees)

(Focus group 1)

Technological solutions were highlighted as promising, including solar panels on buildings to capture heat and cooling systems for streets, inspired by practices from warmer regions. These measures were framed as essential for creating better conditions for older adults to sustain outdoor activities during periods of extreme heat.

Three recurring elements in participants’ reasoning were water, shade, and seating—identified as crucial for a functional and health-promoting urban outdoor environment during heatwaves. Fountains should be operational and accessible for cooling, for instance, by allowing individuals to dip their feet or simply enjoy the sound of flowing water. Seating was also emphasized, with calls for strategic placement in shaded areas such as beneath trees or pergola roofs, contrasting with current arrangements where benches are often positioned directly in the sun.

### Opportunities and Barriers in the Urban Outdoor Environment

Participants’ perspectives highlighted the ambivalence embedded in the physical and social environment that could both facilitate and hinder everyday activities for older adults during heatwaves. The same location could be experienced as simultaneously supportive and limiting. Public transport was similarly framed as both enabling and constraining, revealing its dual role as resource and risk. These tensions are further explored in the subthemes *Two sides of the same place* and *Public transport—a double-edged resource*.

#### Two Sides of the Same Place

Certain urban and nearby locations were framed as “oases” during heatwaves, valued for their water, greenery, shade, and functional seating. Yet this sense of refuge was tempered by frustration over accessibility as narrow paths, sandy beaches, and uneven surfaces exclude those using mobility aids. However, ramps at some bathing sites were mentioned as how small design choices can shift experiences from limitation to inclusion. As previously mentioned, adjusting outdoor activities to evening hours was seen as a strategy to avoid the most extreme heat, yet this adaptation entailed its own cost. Feelings of insecurity in the urban environment at night curtailed opportunities for engagement, and participants voiced a desire for safer evening settings. The paradox of vegetation was repeatedly emphasized: large bushes offered welcome shade while at the same time provoked unease by obstructing visibility, underscoring the conflicting demands placed on outdoor spaces during heatwaves. This tension was elaborated in one discussion:

Then you need to create safe evening environments, I think (group agrees). If people are going to go out around 11 or 12 at night, when it’s really cool.

Yes, when it’s cooler.

Then you have to be sure the environment is safe.

Yes, otherwise it doesn’t work.

I think that’s the most important thing. And much more greenery.

Yes, but now they make sure the bushes aren’t too tall, so people can’t hide in them.

They’ve done a lot in Lasarettsparken, you can’t hide there anymore.

But then there’s no shade either.

Exactly. (group agrees)

(Focus group 1)

These reflections illustrate how safety and comfort were intertwined, with participants acknowledging that measure to reduce insecurity, such as trimming vegetation, could simultaneously diminish shade and cooling. The exchange highlights the ambivalence embedded in urban design, where older adults’ needs for both visibility and shelter were often in tension. Places offering seating and social interaction were considered important during heatwaves, yet participants expressed disappointment that such areas were often inaccessible in practice as they were often occupied by other groups such as youth gangs or individuals with substance use issues. This dilemma added another layer to the experience of insecurity among older adults in outdoor urban settings. Participants expressed that there is a lack of seating specifically designated for older people but viewed this as a political issue rather than something they could influence directly.

#### Public Transport—A Double-Edged Resource

Public transport was portrayed with ambivalence, simultaneously as a lifeline and a potential hazard during heatwaves, with reflections primarily focused on city buses and trams. At times, participants described public transport as a refuge and an opportunity to travel around the city, observe the surroundings, reach summer destinations, or even escape the heat of the urban environment. Discounted ticket options for older adults reinforced this sense of accessibility and inclusion. Yet these positive accounts were tempered by frustration and concerns, because without air-conditioning, vehicles became unbearably hot and were perceived as dangerous, transforming mobility into risk. This ambivalence was raised in all focus groups with an example here:

One thing I really appreciate is that if the bus air-conditioning works, it’s very cool inside. Then you can ride around and look at the surroundings.

Yes, mm. (laughter and agreement from the group)

I’ve found that really good. Not all of them work, but some buses are very cool in the summer. (group agrees) I think that’s great - you can take a trip to Gränna.

Yes, if you buy one of those cheap passes like we pensioners do, then you can do that.

Yes, I do that.

It’s really great.

(Focus group 3)

These reflections illustrate how public transport was simultaneously valued as a cooling refuge and a means of social participation, while also revealing the fragility of this resource. The benefits depended on functioning air-conditioning, where the absence of cooling was transformed into a risk and a significant barrier to mobility and engagement in desired outdoor activities.

## Discussion

The results demonstrate that several interacting dimensions influence older adults’ activities in urban outdoor environments during heatwaves. A recurring pattern was the variation in personal and environmental conditions shaping how older adults experienced and responded to activities, underscoring that they should not be regarded as a homogeneous group. This aligns with the transactional perspective on occupation, which emphasizes the dynamic interaction between individuals, environments, and temporal conditions [26]. The results therefore point to the relevance of a holistic and transactional approach in the development of Age-Friendly Cities and Communities [18], highlighting the need to account for climate-related challenges such as heatwaves. Occupational therapists can contribute valuable expertise by translating this complexity into knowledge about environmental factors that influence opportunities for outdoor activities during heatwaves. The present study may serve as an inspiration for such efforts.

To promote activity and participation in society among older adults, urban outdoor environments need to be designed with attention to accessibility, safety, and inclusivity [27], which aligns with the results. The WHO’s global initiative on Age-Friendly Cities and Communities places a responsibility on all nations to support independence, inclusion, and participation [11]. This responsibility includes ensuring that older adults’ everyday lives during heatwaves are not treated as an exception. In the Nordic region, however, guidance on how to address the emerging challenges of more frequent and intense heatwaves remains limited. The results revealed uncertainty about the future and highlighted the importance of learning from experiences in southern Europe. Cities could be further developed with a stronger focus on everyday activities and participation in society, and occupational therapists may play an important role in decision-making processes related to urban design [28]. Participants in the present study emphasized the importance of involving older adults in decisions regarding urban adaptations for heatwaves. This aligns with previous research suggesting that occupational therapists may contribute by facilitating citizen participation, advocating for age-friendly environments, and bringing knowledge of how environmental conditions influence everyday activities and participation in society [28].

Social dimensions were central in the results. Outdoor activities are essential for older adults’ health and for preventing social isolation [16]. The results emphasized the risk of social isolation during heatwaves, which is supported by Kafeety et al. [29], who argue that this issue should be given greater weight when assessing the negative health impacts of extreme heat. Social networks provide companionship, encouragement, and practical support, and neighborhood cooperation emerged as an important resource, consistent with previous research [15, 18, 28, 29]. Long-term strategies are needed to ensure the continuity of social activities during summer months as heatwaves are becoming more frequent.

Emergency response preparedness also emerged as an important aspect in the results. Although heat warning systems are essential [14, 30], many countries’ systems fail to reach all individuals due to limited communication channels [31]. In Sweden, warnings primarily target risk groups via the healthcare system and social services [2], leaving many older adults without access to timely information. The results point to alternative communication channels and clarify the type of information older adults wish to receive, offering decision support for improving existing systems. Preparedness plans for extreme heat conditions should be locally adapted [14] and include guidance on places older adults can seek out or avoid in their immediate surroundings.

Additional preparedness measures identified in the results included cooling facilities and access to water. Such interventions reduce health risks during heatwaves [14] and are particularly relevant for older adults [15]. The results also pointed to future solutions, such as underground shelters or repurposed bus depots. Such cooling centers have been described not only as places for heat relief but also as spaces that foster social interaction and counteract isolation [29]. The importance of access to water was further underscored in the results, aligning with earlier research [4, 14, 15]. Jay et al. [14] argue that cooling is more effective when water is in motion rather than stagnant, which supports the need for public fountains in urban areas. The results also highlighted simple strategies such as foot immersion, which have been shown to reduce cardiovascular strain, improve thermal comfort, and decrease dehydration in hot climates [14]. Such measures should therefore be made more accessible and integrated into heat warning systems and recommendations.

Shade was identified as the most important factor for enabling activities in urban outdoor environments during heatwaves, with proposed solutions including large trees, pergola roofs, and sun sails. Elevated canopy structures and appropriately designed tree crowns can create cool urban islands [14]. Increased tree canopy coverage reduces the likelihood of older adults avoiding outdoor activities [4], yet tree coverage is often deprioritized in new urban developments [32]. Considering tree coverage as a long-term health-promoting strategy aligns with the principles of Age-friendly urban planning.

Finally, indoor environments and public transport were included in the results because they influenced older adults’ ability to engage in outdoor activities. Housing conditions shaped how older adults related to being outdoors and their ability to recover after exposure to heat. Public transport was highlighted as an alternative to outdoor activity and a means of being mobile during heatwaves. This supports the notion that transport is a defining feature of Age-Friendly Cities and Communities [33]. The ability to move within one’s local environment is a key aspect of maintaining freedom, accessing various places, and preserving roles and routines, as emphasized in the WHO’s global guide to Age-Friendly Cities and Communities [34]. These aspects are inseparable from outdoor activity and align with the transactional perspective on occupation [17, 26], in which all factors are integrated into the reasoning about older adults’ activities in urban outdoor environments during heatwaves and mutually influence one another over time.

## Conclusion

The results provide insight into how older adults reason about activities in urban outdoor environments during heatwaves across individual, group, and societal levels. At the individual level, the importance of personally adapted strategies was evident, with established routines serving as a central foundation for participation in outdoor activities. Heatwaves sometimes necessitate adjustments in habitual patterns which, according to the results, may present challenges. Furthermore, social networks were highlighted as crucial for enabling outdoor activity during periods of extreme heat. At the group level, considerable variation was apparent within the target population, as outdoor activity is influenced by both personal and environmental conditions, underscoring the need to understand older adults as a heterogeneous group. At the societal level, the importance of designing urban outdoor environments to meet the specific challenges posed by heatwaves was emphasized, along with the need for locally adapted heat warning systems tailored to the needs of older adults. This requires both short-term measures and long-term strategies to promote heat-adapted Age-Friendly Cities and Communities. A key, overarching conclusion is that the factors identified at different levels cannot be viewed in isolation but must be understood as dynamically interacting. The results clearly demonstrate that opportunities and barriers in the outdoor environment are affected by a complex interaction between individual capacities, social structures, and societal conditions. This highlights the need for a holistic perspective in the planning of Age-Friendly Cities and Communities to foster participation, autonomy, and resilience among older adults in the face of climate change.

### Significance

The results support the role of occupational therapists in preventive initiatives to promote activities among older adults in urban outdoor environments during heatwaves. For example, the results suggested that municipalities should be able to offer temporary home care assistance during periods of extreme heat. Kafeety et al. [29] argue that municipalities should engage in pre-season planning to identify persons at risk of social isolation, enabling faster provision of support during heatwaves. Through a holistic understanding of occupational performance and environmental conditions, occupational therapists can play a key role in identifying older adults at risk of limited participation in their local community or deteriorating health during heat events. Risk identification cannot be based solely on physical function but requires a comprehensive assessment of the person’s capacities, environment, and meaningful activities. Older adults at risk need support to maintain and adapt their activities in response to changing conditions during heatwaves.

The results also emphasize the importance of habits during heatwaves, including the need to possess, preserve, and modify routines. Habits are central to occupational performance but are dependent on the stability of the surrounding environment [35]. This means that changes in climate, such as heatwaves, or alterations in the urban landscape inevitably affect habitual patterns. Habits can be difficult to change, and the process of change takes time. Many older adults have well-established activity patterns, making adaptation even more challenging, and habit changes may be experienced as disruptive [35]. Occupational therapists can support individuals in adjusting their routines to new conditions, such as those brought on by heatwaves. Interventions must occur at the individual level, as habits are shaped by personal context and specific needs. However, a physical and social environment at the societal level is also required to accommodate these new habits, as the environment at that level can either facilitate or hinder change.

A particular challenge in habit changes related to heatwaves is that new routines may only be temporary during the hot season and not become lasting adaptations. The longer heatwaves persist, the greater the risk that older adults become entrenched in temporary patterns. The results included comparisons to the COVID-19 pandemic, during which many older adults became inactive and increasingly dependent on their immediate surroundings. The role of occupational therapists in supporting older adults’ engagement in urban outdoor activities is therefore equally important before, during, and after heatwaves—at the individual, group, and societal levels.

## Methodological Considerations

The scientific quality of the present study is discussed in relation to reflexivity, theoretical grounding, transparency, and internal coherence [25], and the reporting follows the Reflexive Thematic Analysis Reporting Guidelines (RTARG) to support methodological clarity and openness [36]. In addition, the concepts of credibility, confirmability, and transferability [37] are applied, interpreted in accordance with the study’s reflexive and social constructivist approach. Within this framework, these concepts are not understood as objective measures, but rather as expressions of openness, clarity, and depth in the research process.

The choice of focus groups as a method was considered appropriate for addressing the study’s aim, as it enabled the existing breadth and variation in participants’ experiences, perceptions, and reflections to be articulated and explored [19]. This methodological choice aligns with the study’s social constructivist theoretical framework and was intended to foster rich discussions that could deepen the understanding of older adults’ activities in urban outdoor environments during heatwaves. An identified limitation of the method is that it primarily captures verbal reasoning in group settings. Future studies could strengthen the approach through methodological triangulation by combining focus groups with complementary methods, such as observations of older adults in urban outdoor environments or survey studies. Such an approach may broaden the range of perspectives represented and provide additional insights into everyday adaptations during heatwaves [38].

The study sought credibility by including participants with diverse backgrounds, aiming to capture a wide range of perspectives [19]. Despite this, the participants constituted a relatively homogeneous group and described themselves as the “healthier segment of the older population.” The strategic sampling, based on voluntary participation and recruitment through social meeting places, limited the variation in gender, health status, and life circumstances. The predominance of women in the participant group may have influenced the perspectives represented in the findings. A broader recruitment strategy could have included older adults with disabilities or social vulnerability, which might have enriched the results. Furthermore, all focus groups were conducted in cities located in environments near water, which may have influenced participants’ experiences and thus limit the transferability of the results. Including cities with a more pronounced inland climate could have strengthened this aspect.

Credibility and confirmability were enhanced by concluding each focus group with a summary, allowing participants to confirm, correct, or reject parts of the summary [37, 39]. The reflexive approach throughout the process contributed to awareness of how preunderstanding could influence both analysis and interaction with participants. After each focus group, reflective discussions were held between the participating authors, forming the basis for continuous adjustments for the following focus groups [19]. One example was the term “outdoor activity,” which in the first focus group was interpreted differently, where occupational therapists associate it with all forms of everyday activity, while participants primarily linked it to specific leisure pursuits, such as playing boule. This insight was considered in subsequent groups to avoid conceptual confusion.

Transparency in the study was strengthened through careful documentation of the data collection, including audio recordings, transcription, and field notes. This facilitated the reflexive, analytical process and increased the traceability of the study’s progression. The thematic development demonstrated internal coherence, and excerpts from the discussions were presented to support the interpretations. Credibility might have been further enhanced if more individuals had participated in the analysis, as investigator triangulation can strengthen trustworthiness [39], but ongoing reflective dialogue with the co-authors contributed to analytical depth. Two of the authors participated in and jointly conducted all focus groups, which likely strengthened both the depth of analysis and its contextual grounding. This is consistent with Braun and Clarke [25], who emphasize that having the same individuals responsible for both data collection and analysis can be a strength, as it increases contextual sensitivity.

Since the focus groups were conducted in early spring and participants had not recently experienced extreme heat, the use of prompting was deemed justified. The prompting in the form of images, newspaper headlines, and video clips was used in the beginning of the sessions to evoke memories of previous heatwaves. Such material can be an effective way to stimulate conversation but may also influence participants’ associative pathways and limit variation in perspectives [40]. It can also introduce some recall bias, as memories from previous summers may be incomplete or selective [41]. Therefore, the purpose of the material was carefully considered [19].

## Data Availability

Data from this study can be made available upon request and in accordance with applicable laws.
